# A multidimensional model of healthy ageing: proposal and evaluation of determinants based on a population survey in Ecuador

**DOI:** 10.1186/s12877-021-02548-5

**Published:** 2021-11-01

**Authors:** María Fernanda Rivadeneira, María José Mendieta, Jessica Villavicencio, José Caicedo-Gallardo, Patricio Buendía

**Affiliations:** 1 Pontificia Universidad Católica del Ecuador, Facultad de Medicina, Instituto de Salud Pública, Quito, Ecuador; 2 Department of Public Health, Institute of Nursing Science, University of Basel, Basel, Switzerland; 3 Department of Public Health and Primary Care, Patient Related and Public Health Research, KU Leuven, Doctoral School of Biomedical Sciences, Leuven, Belgium; 4 Pontificia Universidad Católica del Ecuador, Facultad de Medicina, Posgrado de Geriatría y Gerontología, Quito, Ecuador

**Keywords:** Healthy ageing, Quantitative research methods, Multidimensional approach, Cross-sectional study

## Abstract

**Background:**

Healthy ageing is a complex construct which involves multiple dimensions. Previous studies of healthy ageing have focused only on measuring the intrinsic capacity of the older person. The objectives of this study were to design a multidimensional model of healthy ageing and to identify its determinants from national data in Ecuador.

**Methods:**

A cross-sectional analytical study was carried out from the National Survey of Health and Well-being of the Older Adult, 2010. Sample was 1797 adults aged 65 years or more. A multidimensional model was designed based on the World Health Organization’s concept of healthy ageing. For the analysis, two groups were created: a *healthy ageing* and a *less healthy ageing group*. Bivariate and multivariate logistic regressions were performed to analyze the probability of belonging to the healthy group according to sex, age, area of ​​residence, level of education, perceived health status, perceived life satisfaction, and poverty by income level.

**Results:**

The 53.15% of the sample was classified in the healthy ageing group. Women and the poorest older adults were less likely to be in the healthy ageing group (OR 0.58; 95% CI 0.464–0.737; OR 0.44; 95% CI 0.343–0.564). Older adults with secondary education or higher, who considered their health as excellent and who were satisfied with their life, had a greater probability of being in healthy ageing group (OR 2.61; 95% CI 1.586–4.309; OR 28.49; 95% CI 3.623–224.02; OR 0.23; 95% CI 0.165–0.341).

**Conclusions:**

This study contributes with a multidimensional approach to healthy ageing. It proposes to evaluate the intrinsic capacity of the individual, the social and political environment and the interaction with it, through indicators that discriminate who are ageing in a healthy way and who are not. By using this model, it was identified that gender and economic situation seem to play an important role on heathy ageing of the Ecuadorian population. Public policies are necessary to promote healthy ageing, especially focused on improving socioeconomic conditions and gender equity.

**Supplementary Information:**

The online version contains supplementary material available at 10.1186/s12877-021-02548-5.

## Background

The ageing of the population represents a challenge for the health and social care systems. Globally, the number of people over 60 has increased alarmingly in the last 30 years and it is expected that by 2030, they will exceed the number of children under the age of ten [1]. Multiple chronic conditions affect this population, impacting negatively in their quality of life and increasing their use of health and social care services [2]. Interventions aimed at preventing diseases and disabilities have proven to be cost-effective over time [3], so promoting healthy ageing in the population is appointed as the best strategy. However, until now there is no consensus around its conceptualization and how to measure it [4].

The efforts for associating a positive concept to the ageing process are not a recent endeavor. In the forty-fourth century BC, Cicero described ageing as a time of opportunities for positive change and productive functioning, while in the 1940s the use of the *successful ageing* concept was linked to the first attempts to develop indicators to determine the degree of satisfaction with life [5]. Later, Havighurst adopted it, describing the *successful ageing* as an adaptive theory and a testable experience [6]. But it was Rowe and Kahn [7] who popularized it to make a differentiation between “usual or normal” ageing from “successful” ageing. According to them, *successful ageing* encompasses older adults who have a low probability of disease and disability, high physical and cognitive capacity and an active commitment to life [7]. This conceptualization has been widely criticized and discarded for its restrictive approach [4–8].

Other attempts to describe the ageing process from a positive perspective have been done in Europe, by introducing the concept of *active ageing* [9]. “Active” was defined as a continuous participation in social, economic, cultural, spiritual and civic aspects and not only the ability to stay physically active or participate in the workforce [9]. But this model has not been validated either. Paúl, Ribeiro and Teixeira [10] demonstrated that most of the determinants of this model were not independent; while Bélanger and colleagues [11] concluded that the active ageing concept could be considered as a human rights policy orientation rather than an empirical measurement tool. Other authors have pointed out the risk of discrimination when idealizing the concept of active ageing, as a status that the entire older population should reach [9].

The *healthy ageing* term has been used at academic and political levels, to make a distinction between ill and not ill older adults, based mainly on their physical and mental attributes [4, 5]. In the United States, McLaughling, Jette and Connell [12] identified a prevalence of healthy ageing ranging from 3.3 to 35.5%. They used four operational definitions of healthy ageing based on the Rowe and Kahn definition of successful ageing. The authors highlighted that the large variation in the prevalence was the consequence of using rigid criteria, suggesting that greater importance should be given to the presence of symptomatic disease or the impact that it has on the functional status of the older person [12]. Similar results were found in a study of the Spanish population, with a prevalence of healthy ageing ranging from 4.5 to 49.2%, depending on the criteria used to define it [4]. Recently, Dieteren and colleagues [13] published results of the ageing trajectories of a cohort of older adults, using the Healthy Ageing Index (HAI). The HAI predicts mortality, independently of the age or the presence of comorbidities, based on five physiological indicators. The authors highlighted the differences in the ageing trajectories, based on sex, nutrition and physical activity to achieve an optimal ageing [13].

In 2015, the World Health Organization (WHO) provided a more holistic definition of healthy ageing, defining it as “the process of promoting and maintaining functional capacity that allows well-being in old age.” [14]. It is based on the interrelation between the intrinsic capacity of a person and their environment. Intrinsic capacity refers to all the mental and physical capacities that a person can rely on, including their ability to walk, think, see, hear, and remember. The environment includes interpersonal factors, such as the relationships that the older adult develops with other people and their participation in the community, contextual factors such as access to services, existing health and social policies, and the physical environment [14].

The aim of this study was to develop a multidimensional model of healthy ageing that incorporates measurements of the *intrinsic capacity* of the individual, the *social and political environment,* and the *interaction with the environment*. Additionally, we identified the determinants associated with healthy ageing, based on data from a national survey from Ecuador.

## Design and methods

Analytical cross-sectional study with a secondary database from the National Survey of Health and Well-being of the Older Adult, 2010 (SABE, 2010).The SABE survey was carried out by the National Institute for Statistics and Census (INEC) and the Ministry of Economic and Social Inclusion (MIES) to identify the health and nutrition conditions of people over 65 years in Ecuador. The sample was random, probabilistic, and three-staged, proportional to the size of the population [15]. It was representative for the population over the age of 65, according to the 2010 Census of INEC. The sample was limited to 1797 observations, corresponding to 898,152 adults aged 65 and over, after applying the expansion factor -suggested by the INEC to expand the sample to population size in 2010-. Survey data is available online at: https://anda.inec.gob.ec/anda/index.php/catalog/292 [16].

### Components and domains of the healthy ageing model

**Fig. 1:**
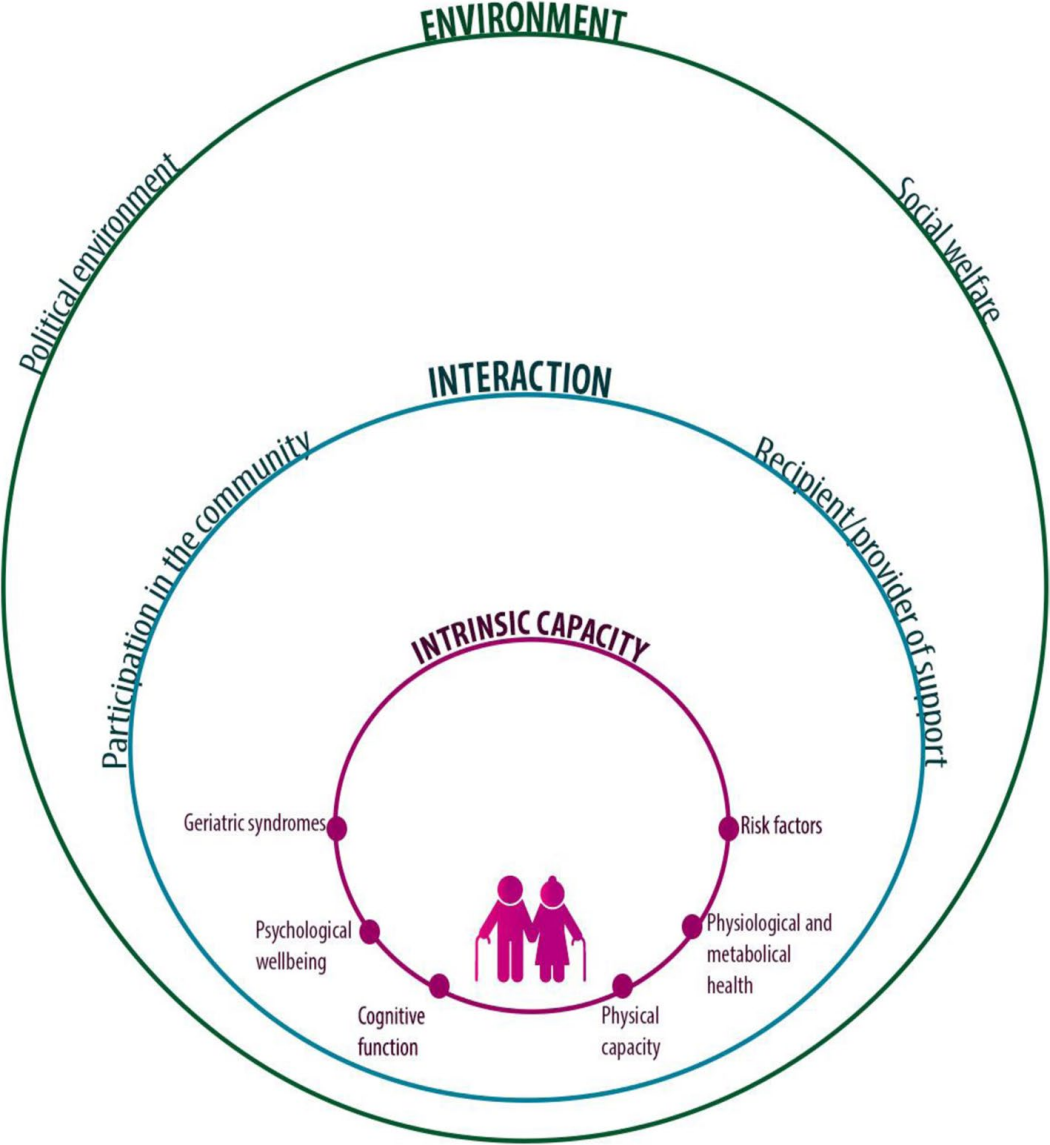
Multimensional model of healthy ageing. The graph summarizes the components of the proposed multidimensional model: **a** intrinsic capacity [in red], **b** social and political environment [in green] and **c** interaction of the older adult with the environment [in blue], which determine the functional capacity of the elderly

A healthy ageing model was developed based on the WHO concept of healthy ageing [14]. Three components were considered: a) the *intrinsic capacity*, referring to the physical and mental health, b) the *social and political environment*, and c) the *interaction of the older adult with the environment*. To determine the intrinsic capacity, six domains were considered: 1) *physiological and metabolic health*, 2) *geriatric syndromes*, 3) *risk factors*, 4) *physical capacity*, 5) *cognitive capacity*, and 6) *psychological well-being* (Fig. 1). The *physiological and metabolic health* domain considered whether the individual has/had cancer, or has diabetes, hypertension or other chronic diseases, but they are under treatment or do not limit the functional status of the older person. The g*eriatric syndromes domain* includes the absence of urinary and fecal incontinence, the number of falls and the presence of polypharmacy. In the *risk factors domain* we included measurements for cardiovascular risk, consumption of alcohol, tobacco, and physical activity. The *physical capacity domain* included compliance with activities of daily living, and a self-reported assessment of mobility, while the *cognitive ability domain* involved an assessment of dementia or cognitive impairment. The absence of physical, sexual, and psychological abuse, as well as absence of depression, where included in the *psychological well-being* domain.

The *social and political environment* component considered two domains: 1) the *social welfare* domain, which incorporated the social situation data as well as the presence of negligence or economic violence, and 2) the *political environment* domain, which measured if the older person had access to social security, health services and support from different institutions.

The component of *interaction of the older adult with the environment* involved the following domains: 1) *participation of the older adult in the community*, and 2) *support provided by them and/or received* from children, siblings and other family members or friends.

For each of the domains, criteria were established based on prior knowledge [5, 17–29], to conform two groups: a *healthy group* and a *less healthy group* (Table 1).

**Table 1:** Components, domains and concepts of the healthy ageing model for the older population in Ecuador

Healthy Ageing Components	Domains	Healthy group	Less healthy group
*Intrinsic capacity*	*Physiological and metabolic health*	Absence of the following diagnoses: arterial hypertension, diabetes, cancer, chronic lung disease, cardiovascular disease, cerebrovascular disease, arthritis, rheumatism, osteoporosis and osteoarthritis, others; or the presence any of these diseases, but they are under treatment or they do not limit the functional status of the older person.	With a diagnosis of at least one of the aforementioned diseases, which are not under control, and/or limit the functional status of the older person.
	*Geriatric syndromes*	Absence of polypharmacy [17], urinary and fecal incontinence [18–20] and no falls syndrome [21, 22].	Presence of any: polypharmacy, urinary and fecal incontinence and falls syndrome.
	*Risk factors*	Absence of risk factors for cardiovascular disease such as blood pressure > 150/90, fasting glucose ≥126, obesity (body mass index, [BMI] ≥ 30), no alcohol consumption more than 1 day a week, no current and past tobacco use, physical activity and no hypercholesterolemia according to Adult Treatment Panel III (ATP-III) [23].	Presence of any risk factors for cardiovascular disease: blood pressure > 150/90, fasting glucose ≥126, obesity (body mass index, [BMI] ≥ 30) or presence of at least one of: alcohol consumption more than 1 day a week, current and past tobacco use, no physical activity and abnormal values of lipoproteins and triglycerides [23].
	*Physical capacity*	Meets 6 to 7 of the following: 1) stands with the feet together keeping eyes open, 2) stands with the heel of one foot in front of the other foot, 3) stands on one foot without leaning or holding onto anything, 4) feels able to get up quickly from the chair five times, 5) gets up quickly from the chair five times, 6) feels able to get up from the chair with the arms on the chest five times, and, 7) gets out of the chair with the arms on the chest five times [4]. Absence of disability according to the Katz activities of daily living (ADL) test [24].	Meets 5 or less of the above instructions. Presence of disability according to the Katz activities of daily living (ADL) test [24].
	*Cognitive ability*	Absence of dementia and cognitive deficit. Cognitive ability was obtained from the modified Mini-mental assessment. A score ≥ 14 was used as a cut-off point for the absence of cognitive deficit [25].	Presence of dementia and/or cognitive deficit. A score < 13 in the Mini-mental assessment was used to define cognitive deficit [25].
	*Psychological well-being*	Absence of physical, sexual or psychological abuse collected by directly asking the older person. Absence of depression by using of the Yesavage geriatric depression scale [26–28].	Presence of any of following: -Physical, sexual or psychological abuse. -Depression (≤ 4 in the Yesavage scale) [26–28].
*Social and political environment*	*Social welfare*	Absence of negligence, and/or economic violence. A score of ≤9 in the Gijón scale, was defined as a good / acceptable social situation [29].	Presence of negligence, and/or economic violence. A score of > 10 in the Gijón scale [29].
	*Political environment*	An older person beneficiary of social security or receiving a non-contributory pension, and having access to health services, as well as receiving support from different institutions.	An older person who does not have social security, nor receive a non-contributory pension. Does not have access to health services, as well as recipients of support from different institutions.
*Interaction of the older person with the environment*	*Participation in the community*	An older person participating in recreational activities, and acting as volunteer in an institution or organization in the community at least once a month [5].	An older person who does not participate in recreational activities, neither acts as volunteer in an institution or organization in the community.
	*Recipient or provider of support*	An older person receiving or providing support from children, siblings and other family or friends.	An older person who does not receive or provide support from children, siblings and other family or friends

### Statistical analysis

**Fig. 2:**
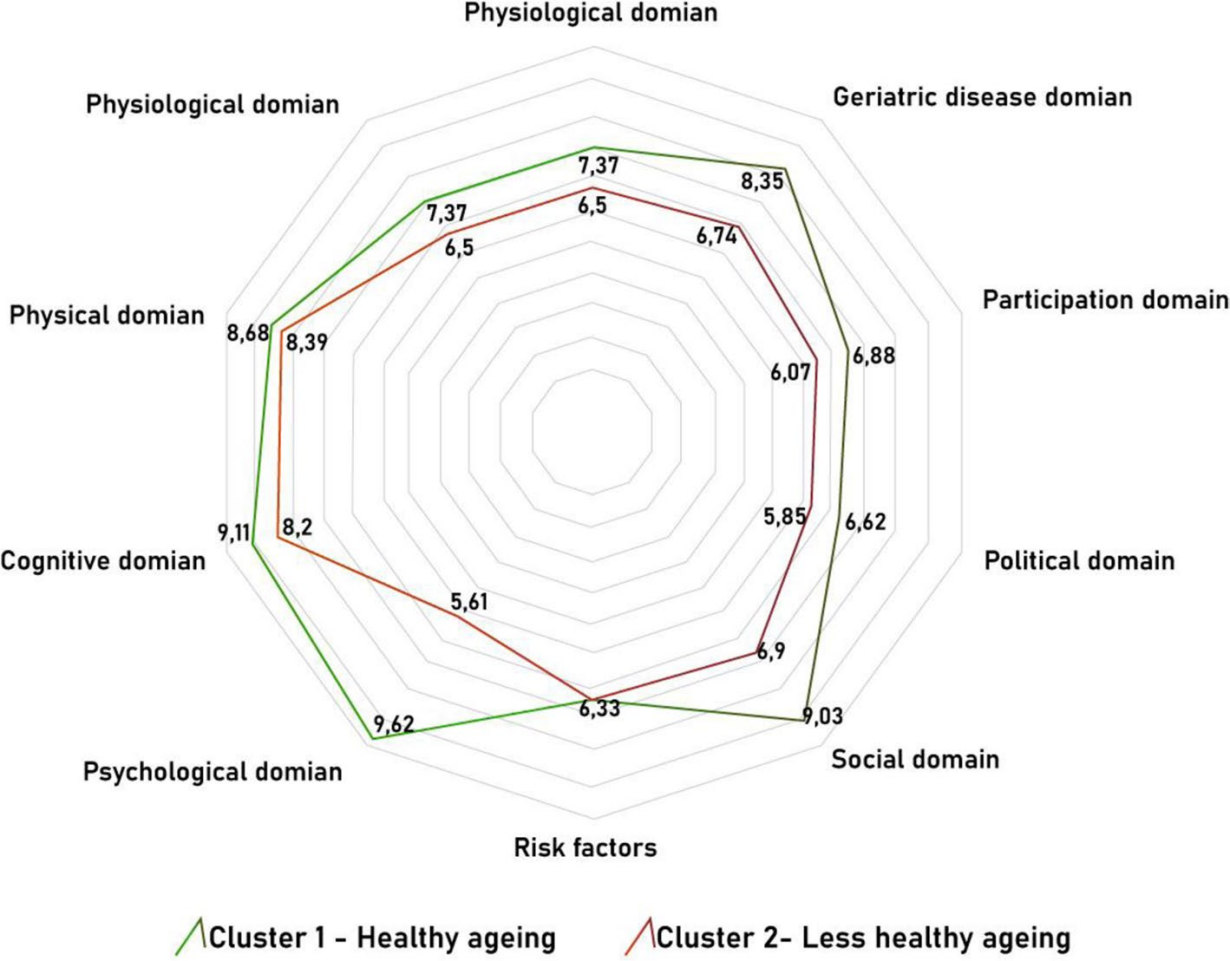
Radar chart showing two clusters with domains of the healthy ageing model. The graph presents two clusters: cluster 1 – “the *healthy ageing group*”, and cluster 2 – “the *less healthy group*”. The number represents the health status of each domain, the closer it is to 10, the better health status according to each domain

Based on the proposed theoretical model (Table 1), variables from the SABE survey were selected using the experience of previous studies focused on determining the prevalence of healthy ageing, as well as the expertise of the researchers [4, 12]. From these variables, two theoretical groups were defined a priori: a healthy ageing group and a *less healthy ageing group* (Additional file 1). A two-stage cluster subdivision into intra-domain categories was performed according to compliance with the theoretical model, where the highest number reflects a better health status with respect to each domain, being 10 the highest score. Then, using a two-stage cluster categorization process, people were grouped into two clusters according to the similarity of their inter-domain categories. A radar graph was designed with the means of the domains for each cluster. It showed better scores for cluster 1 (healthy ageing group) than for cluster 2 (less healthy ageing group) (*p* value < 0.05), except in the *risk factors domain*, where a significant mean difference was not found (Fig. 2).

Finally, a bivariate logistic regression for complex samples, with an expansion factor available at the SABE dataset, was performed with the dependent variable of being part of the *healthy ageing group.* Then, with the significant variables (*p* < 0.05), a multivariate logistic regression for complex samples was performed. Explanatory variables were age, sex, area of ​​residence, level of education, perceived health status, perceived life satisfaction, and poverty by income level. Statistical programs STATA® version 15.1, and SPSS® from IBM® version 25 were used for the statistical analysis.

## Results

In the analyzed data, 56.65% were women and 43.35% men. More than half of the individuals were in the range of 65 to 74 years old (58.47%). Complete information on the demographic characteristics of the sample is detailed in Table 2.

**Table 2:** Demographic and socio-economic characteristics, older adults included in the study. National SABE-Ecuador survey, 2010–2011

Variable	Frecuency (n°)	Relative Frequency (%)
Gender		
Woman	508,876	56.66
Man	387,285	43.12
Missing values	1991	0.22
Age	1991	0.22
65–74 years	475,053	52.89
75–84 years	261,416	29.11
85 years or more	76,046	8.47
Missing values	85,637	9.53
Area		
Urban	584,700	65.1
Rural	313,452	34.9
Missing values	0	0
Ethnic self-identification		
Mestizo^a^	575,297	64.05
White	118,342	13.18
Indigenous	93,643	10.43
Mulatto^b^	30,569	3.4
Afro ecuadorian	30,171	3.36
Other	12,600	1.4
Missing values	37,529	4.18
Level of education		
None	15,181	1.69
Literacy center	19,632	2.19
Kinder Garden	2745	0.31
Primary	503,793	56.09
High school	82,704	9.21
College	23,778	2.65
Post graduate	2254	0.25
Missing values	248,065	27.62

^**a**^Mestizos = ethnic group born from the cross between Europeans, indigenous and Africans during the colonial era. It is a hegemonic category that includes other socio-racial identities, including indigenous people themselves and black people with all their derivations

^b^Mulatto = relating to black people of African descent mixed socio-racially with white or mestizo populations

According to previously established criteria and cluster analysis, 53.15% of the sample was grouped into the *healthy ageing group*, and 46.85% corresponded to the *less healthy ageing group*. Table 3 describes the characteristics of these groups. The *healthy ageing group* showed a higher proportion of men than women (60.74% vs 47.54%, respectively), younger older adults (57.41% between 65 to 74 vs 31.18% of 85 years old and older), living in urban areas (55.74%) rather than in rural areas (47.47%), mestizos (56% vs 35% of indigenous) and older adults with higher education (89.62% with college vs 41.07% without formal education). Regarding perceived health status, 91.46% of older adults, who considered their health as excellent, and the 58.62% who were satisfied with their life, were in the healthy ageing group. In this group, 61.7% of the older adults were not poor.

**Table 3:** Socio-demographic characteristics for healthy and less healthy ageing. National Base SABE-Ecuador, 2010–2011

Variable	Healthy ageing (n)	Relative frequency (%)	Less healthy ageing (n)	Relative frequency (%)
Gender				
Man	200,574	60.74	129,623	39.26
Woman	205,012	47.54	226,230	52.46
Age				
65–74 years	239,846	57.41	177,899	42.59
75–84 years	117,437	51.29	111,549	48.71
85 years or more	16,204	31.18	35,773	68.82
Area				
Urban	292,002	55.74	231,828	44.26
Rural	113,750	47.47	125,850	52.53
Ethnicity				
Mestizo	280,883	56.00	220,681	44.00
White	53,999	51.47	50,923	48.53
Indigenous	23,955	35.02	44,451	64.98
Afro ecuadorian	15,452	64.69	8436	35.31
Mulatto	14,900	56.64	11,408	43.36
Other	4367	38.50	6977	61.50
Level of eduction				
None	4903	41.07	7036	58.93
Literacy	4522	26.01	12,862	73.99
Kinder garden	1212	44.15	1533	55.85
Primary	240,836	54.82	198,513	45.18
High school	58,032	76.03	18,293	23.97
Bachelor	1717	100.00	0	0.00
College	18,143	89.62	2102	10.38
Post Graduate	2254	100.00	0	0.00
How do you consider your health				
Excellent	7180	91.46	670	8.54
Very good	14,484	76.54	4440	23.46
Good	117,367	73.79	41,697	26.21
Regular	224,147	53.24	196,861	46.76
Bad	42,574	27.33	113,200	72.67
Are you satisfied with your life				
Yes	373,827	58.62	263,883	41.38
No	30,986	25.18	92,084	74.82
Income poverty^a^				
No	272,076	61.67	169,120	38.33
yes	133,676	41.48	188,558	58.52

^a^Income poverty = income less than USD 1.90 per day

Table 4 shows the results of the bivariate and multivariate analysis for *healthy ageing group*. Women were less likely to belong to the *healthy ageing group*, compared to men (OR 0.58, 95% CI 0.46–0.74), this association remained significant in the multivariate analysis (OR 0.69, 95% CI 0.48–0.93). With regard to age, the group over 85 years was less likely to be in the *healthy ageing group* compared to people between 65 to 74 years (OR 0.33, 95% CI 0.21–0.55); this association remained significant in the multivariate analysis (OR 0.24, 95% CI 0.14–0.41). An older adult living in rural areas was less likely to be in the *healthy ageing group* (OR 0.71, 95% CI 0.54–0.95); this association lost significance in the multivariate analysis. Older adults with secondary education or higher had 2.61 times the probability of being in the *healthy ageing group* (95% CI 1.59–4.31), compared with primary education, and this association remained significant in the multivariate analysis (OR 2.22, 95% CI 1.16–4.23). Older adults who considered their health as excellent had a greater probability of being in the *healthy ageing group*, compared to those who considered their health as poor (OR 28.49, 95% CI 3.62–224.02). Those who were not satisfied with their lives were less likely to be in the *healthy ageing group* (OR 0.23, 95% CI 0.17–0.34). Older adults within the limits of poverty were less likely to be in the *healthy ageing group* (OR 0.44, 95% CI 0.343–0.564). These variables maintained their significant association in the multivariate analysis (OR 14.39, 95% CI 2.92–70.82; OR 0.30, 95% CI 0.19–0.49; OR 0.61 95% CI 0.42–0.89) (Table 4).

**Table 4:** Association between *healthy ageing group* with determinants. SABE-Ecuador national base, 2010–2011

	Bivariate analysis			Multivariate analysis		
Variable	Odds Ratio	95% IC	***p***-Value	Odds Ratio	95% IC	***p***-Value
Gender						
Man	Reference			Reference		
Woman	0.58	0.46–0.78	0.00**	0.69	0.48–0.93	0.01*
Age						
65–74 years	Reference			Reference		
75–84 years	0.78	0.58–1.05	0.09	0.88	0.59–1.30	0.53
85 years or more	0.33	0.21–0.55	0.00**	0.24	0.14–0.42	0.00**
Area						
Urban	Reference			Reference		
Rural	0.71	0.54–0.95	0.02*	0.98	0.67–1.47	0.92
Ethnicity						
Mestizo^a^	Reference			Reference		
Indigenous	1.43	0.72–2.86	0.29	1.06	0.41–2.77	0.91
Afro ecuadorian	1.02	0.48–2.16	0.95	1.28	0.47–3.51	0.63
Mulatto	0.83	0.55–0.13	0.39	0.63	0.39–1.01	0.06
White	0.42	0.27–0.67	0.00**	0.69	0.34–1.39	0.29
Other	0.49	0.19–1.27	0.14	0.29	0.08–1.04	0.06
Level of education						
Primary	Reference			Reference		
None	0.57	0.19–1.72	0.32	0.49	0.14–1.65	0.25
Literacy	0.28	0.11–0.76	0.01	0.28	0.10–0.78	0.02
Kinder gander/ primary	0.65	0.06–7.10	0.72	0.66	0.06–6.71	0.73
High school	2.61	1.57–4.31	0.00**	2.22	1.16–4.23	0.02*
College / post graduate	7.11	2.16–23.39	0.00**	2.85	0.79–10.19	0.11
How do you consider your health						
Bad	Reference			Reference		
Excellent	28.49	3.62–224.03	0.00**	14.39	2.92–70.83	0.00**
Very good	8.67	3.43–21.93	0.00**	7.62	1.64–35.44	0.01*
Good	7.48	5.01–11.17	0.00**	6.61	3.76–11.63	0.00**
Regular	3.02	2.18–4.19	0.00**	3.76	2.50–5.67	0.00**
Are you satisfied with your life						
Yes	Reference			Reference		
No	0.23	0.17–0.34	0.00**	0.3	0.18–0.49	0.00**
Income poverty^a^						
No	Reference			Reference		
Si	0.44	0.34–0.56	0.00**	0.61	0.42–0.89	0.01*

^a^Income poverty = income less than USD 1.90 per day

*Value *p* significant < 0.05; **Value *p* significantt < 0.01

## Discussion

The interest in demonstrating that the ageing process is multidimensional concept is not a novel concern. By using different conceptualizations, many researchers have tried to describe the differences among those who age in better conditions and those who do not. The differences found by McLaughling, Jette and Connell [12] and Rodriguez-Laso and colleagues [4] in the prevalence of healthy ageing in the United States and Spanish population, respectively, highlighted the need to use a more comprehensive criteria to identify the older population who is ageing in a better condition [14].

The present study contributes with a novel multidimensional model to assess the healthy ageing, based on the WHO concept, using a less rigorous definition that incorporates the role of the environment and the interaction of the older adult with it [14]. This comprehensive model breaks the paradigm that being healthy is equal to the absence of physical illness.

According to this model, in 2010, more than half (53.15%) of the population over 65 years old belonged to the group of *healthy ageing* in Ecuador. This result differs from the data reported in the United States and Spain [4, 12]. This can be explained partly due to the sociodemographic differences of the older adults included in the different studies, but particularly by the multidimensional healthy ageing model applied in the present investigation. In our model, the intrinsic capacity of an older person with a healthy ageing was not limited to the absence of a disease or risk factors. As previous studies have shown that, despite physical limitations and structural obstacles, older people can be considered successful or active, given the compensation process they go through as they age [6, 10].

An interesting finding after the two-stage cluster analysis is the similarity of the two groups, healthy and less healthy, in the risk factors domain. This behavior could reinforce the idea that the ageing process and its trajectory does not only depend on the deterioration of the intrinsic capacity of the individual, but also on the environment where the person resides. Previous research has demonstrated that certain risk factors such as: obesity, sedentary lifestyle, smoking, alcohol consumption, have a great impact on how a person ages; but other elements of the environment, including demographic, epidemiological geographical, or economic situation, could affect the ageing trajectory too [13, 30].

In this study, women were less likely to belong to the *healthy ageing group* compared to men. This could be explained due to the association described between high levels of multimorbidity and female sex, where older women show a worse health status when compared to men of the same age [31]. Yet, other authors have pointed out that this association is more linked to age than sex, since it has been seen that after the age of 80 the differences in health status by sex are reduced [6, 30]. In this study, the association between less healthy and female sex was maintained after adjusting for age and other variables, which points to gender inequalities in the way of ageing.

Age, educational level and economic status were also associated with healthy ageing. As a person ages, their risk for suffering from multiple chronic conditions and disability increases, impacting negatively on their intrinsic capacity and consequently in their functional capacity [32–34]. Therefore, it was not surprising that in our study individuals of 85 years old or more showed lower odds of experiencing a healthy ageing. On the other hand, having a higher educational level increased the odds of being in the *healthy ageing group*. These results coincide with previous studies that have shown the positive effect of education in older adults to access to health services, adhere to medical treatments and understand health issues, which in turn contributes to improve their quality of life [35–41].

According to our data, older adults in the worst economic situation were less likely to be in the *healthy ageing group*, making them a highly vulnerable group. There is vast evidence of the association between a low socioeconomic level and poorer physical and mental health in older adults, as a consequence of exposure to a greater number of risk factors, anxiety, and less access to health services from younger ages [40–42].

In the present study, no significant difference was found between living in an urban or rural area. The combination of an unfavorable external environment for the older population, as well as personal poverty increase their risk of having a poor physical, psychological and mental health status, social isolation, and higher risk of death [43, 44]. For this reason, the limitations of the environment, and not simply the place of residence, could be a more important factor to consider when defining whether an older person is ageing in a healthy way or not.

Self-perceived health status and life satisfaction were also associated with healthy ageing. In this study, most of the older adults who had a positive perception of their health were categorized in the *healthy ageing group*. This may be because the *healthy ageing group* includes people with the best intrinsic capacity and an environment that meets their needs. However, this could also be explained in terms of the “disability paradox”; where in presence of severe disability, a person reports high quality of life, due to their ability to adapt to their condition, finding a balance between their intrinsic capacity and the environment [45, 46].

We have some limitations in this study. SABE was conducted 11 years ago, so the reported findings might not reflect the current situation of the Ecuadorian older population. In the last 11 years, Ecuador has experienced important political and socioeconomical changes that could have modified the situation of the older population. However, this is the only survey carried out in the older adult population on a national scale. There is no recent or updated information on the situation of the elderly in Ecuador; therefore it was not possible to perform an analysis of the trend of healthy ageing over time. In the same way, it was not possible to extrapolate the data obtained to the current population, nor to propose a prevalence of healthy ageing, under current conditions. This would require data collection over time, which would be an important recommendation for future studies.

Additionally, the variables included in the domains were restricted to those available in the national survey. Despite the richness of the data, we did not have more in-depth information on laboratory or clinical tests. It is possible that some data contained memory and information biases, particularly data that required a clinical diagnosis, as in the case of previous diseases. On the other hand, due to the characteristics of the study, it was not possible to make cause-effect inferences. Finally, the absence of a unified and operational definition of healthy ageing makes a direct comparison between studies impossible.

The main strength of this study is the design of a multidimensional model of healthy ageing, for which secondary data from a national survey was used. The proposed model offers a holistic approach to healthy ageing, which includes components such as the environment and the interaction of the older adult, which have not previously been addressed. This model contributes not only to understanding the heterogeneity of ageing, but also to identifying the group of older adults who need to be prioritized in public policies. The evidence suggests the importance of healthy ageing policies focused on improving socioeconomic conditions and reducing gender inequalities.

## Conclusion

The model of healthy ageing addresses multidimensional variables in nine domains, which are summarized in three components: the intrinsic capacity, referring to the physical and mental health, the social and political environment, and the interaction of the older adults with the environment. This model conceptualizes healthy ageing in a comprehensive way, discriminating between the healthy group and the less healthy group. By using this model, it was identified that gender and economic situation seem to play an important role on heathy ageing of the Ecuadorian population. Public policies are necessary to promote healthy ageing, especially focused on improving socioeconomic conditions and gender equity.

## Supplementary Information

**Additional file 1 **Theoretical model used for the classification into *healthy* and *less healthy ageing groups.*

## Data Availability

The database for this study is publicly available at Instituto Nacional de Estadísticas y Censos (INEC), Secretaría Nacional de Planificación y Desarrollo (SENPLADES). Encuesta de Salud, Bienestar y Envejecimiento - SABE 2009. 2010. https://anda.inec.gob.ec/anda/index.php/catalog/292 [16].

## Abbreviations

ADL: Activities of daily living
ATP-III: Adult Treatment Panel III
BMI: Body mass index
INEC: National Institute for Statistics and Census
MIES: Ministry of Economic and Social Inclusion
OR: Odds ratio
WHO: World Health Organization
95% CI: 95% Confidence interval

## References

[1] United Nations, Department of Economic and Social Affairs, Population Division. World Population Ageing 2017—Highlights. 2017. https://www.un.org/en/development/desa/population/publications/pdf/ageing/WPA2017_Highlights.pdf. Accessed 5 Jan 2021.

[2] Johnston MC, Crilly M, Black C, Prescott GJ, Mercer SW (2019). Defining and measuring multimorbidity: a systematic review of systematic reviews. Eur J Pub Health.

[3] National Center for Chronic Disease Prevention and Health Promotion. The Power of Prevention: Chronic Disease. The Public Health Challenge of the 21st Century [Data set]. American Psychological Association. 2009. 10.1037/e581002012-001. Accessed 8 Jan 2021.

[4] Rodriguez-Laso A, McLaughlin SJ, Urdaneta E, Yanguas J (2018). Defining and estimating healthy aging in Spain: a cross-sectional study. The Gerontologist.

[5] Bowling A (1993). The concepts of successful and positive ageing. Fam Pract.

[6] Pruchno R (2015). Successful aging: contentious past, productive future. Gerontologist.

[7] Rowe JW, Kahn RL (1997). Successful aging. The Gerontologist.

[8] Martinson M, Berridge C (2015). Successful aging and its discontents: a systematic review of the social gerontology literature. The Gerontologist.

[9] Foster L, Walker A (2015). Active and successful aging: a European policy perspective. The Gerontologist.

[10] Paúl C, Ribeiro O, Teixeira L. Active Ageing: An Empirical Approach to the WHO Model. Curr Gerontol Geriatr Res. 2012;2012:382972. 10.1155/2012/382972 PMC3501803 23193396

[11] Bélanger E, Ahmed T, Filiatrault J, Yu HT, Zunzunegui MV (2017). An empirical comparison of different models of active aging in Canada: the international mobility in aging study. The Gerontologist.

[12] McLaughlin SJ, Jette AM, Connell CM (2012). An examination of healthy aging across a conceptual continuum: prevalence estimates, demographic patterns, and validity. J Gerontol Ser A.

[13] Dieteren CM, Samson LD, Schipper M, van Exel J, Brouwer WBF, Verschuren WMM (2020). The healthy aging index analyzed over 15 years in the general population: the Doetinchem cohort study. Prev Med.

[14] World Health Organization (WHO) (2015). Informe mundial sobre el envejecimiento y la salud.

[15] Freire W, Rojas E, Pazmiño L, Fornasini M, Tito S, Buendía P, Waters W, Salinas J, Álvarez P (2010). Encuesta Nacional de Salud, Bienestar y Envejecimiento. SABE I, Ecuador, 2009—2010.

[16] Instituto Nacional de Estadísticas y Censos (INEC), Secretaría Nacional de Planificación y Desarrollo (SENPLADES) (2010). Encuesta de Salud, Bienestar y Envejecimiento - SABE 2009.

[17] Masnoon N, Shakib S, Kalisch-Ellett L, Caughey GE (2017). What is polypharmacy? A systematic review of definitions. BMC Geriatr.

[18] Chong EC, Khan AA, Anger JT (2011). The financial burden of stress urinary incontinence among women in the United States. Curr Urol Rep.

[19] Ekelund P, Grimby A, Milsom I (1993). Urinary incontinence. Social and financial costs high. BMJ.

[20] Kessler M, Facchini LA, Soares MU, Nunes BP, França SM, Thumé E (2018). Prevalence of urinary incontinence among the elderly and relationship with physical and mental health indicators. Rev Bras Geriatria Gerontol.

[21] Burns ER, Stevens JA, Lee R (2016). The direct costs of fatal and non-fatal falls among older adults—United States. J Saf Res.

[22] Gazibara T, Kurtagic I, Kisic-Tepavcevic D, Nurkovic S, Kovacevic N, Gazibara T, Pekmezovic T (2017). Falls, risk factors and fear of falling among persons older than 65 years of age. Psychogeriatrics.

[23] Cleeman J, ATP III (2001). Guidelines at-A-glance quick desk reference.

[24] Katz S, Downs TD, Cash HR, Grotz RC (1970). Progress in development of the index of ADL. Gerontologist.

[25] Folstein MF, Folstein SE, McHugh PR (1975). “Mini-mental state”: a practical method for grading the cognitive state of patients for the clinician. J Psychiatr Res.

[26] Anderson DN (2001). Treating depression in old age: the reasons to be positive. Age Ageing.

[27] Yesavage JA, Sheikh JI (1986). Geriatric depression scale (GDS): recent evidence and development of a shorter version. Clin Gerontol.

[28] Zis P, Daskalaki A, Bountouni I, Sykioti P, Varrassi G, Paladini A (2017). Depression and chronic pain in the elderly: links and management challenges. Clin Interv Aging.

[29] Alarcón-Alarcón T, González-Montalvo JI (1998). La Escala Socio-Familiar de Gijón, instrumento útil en el hospital general. Rev Española Geriatría Gerontol.

[30] Wong R, Ofstedal MB, Yount K, Agree EM (2008). Unhealthy lifestyles among older adults: exploring transitions in Mexico and the US. Eur J Ageing.

[31] Violan C, Foguet-Boreu Q, Flores-Mateo G, Salisbury C, Blom J, Freitag M, Glynn L, Muth C, Valderas JM (2014). Prevalence, determinants and patterns of multimorbidity in primary care: a systematic review of observational studies. PLoS One.

[32] Hu RH, Hsiao FY, Chen LJ, Huang PT, Hsu WWY (2019). Increasing age- and gender-specific burden and complexity of multimorbidity in Taiwan, 2003–2013: a cross-sectional study based on nationwide claims data. BMJ Open.

[33] Roberts KC, Rao DP, Bennett TL, Loukine L, Jayaraman GC (2015). Prevalence and patterns of chronic disease multimorbidity and associated determinants in Canada. Health Promot Chron Dis Prev Canada Rese Policy Pract.

[34] Barnett K, Mercer SW, Norbury M, Watt G, Wyke S, Guthrie B (2012). Epidemiology of multimorbidity and implications for health care, research, and medical education: a cross-sectional study. Lancet.

[35] Baker DW, Wolf MS, Feinglass J, Thompson JA, Gazmararian JA, Huang J (2007). Health literacy and mortality among elderly persons. Arch Intern Med.

[36] Bennett IM, Chen J, Soroui JS, White S (2009). The contribution of health literacy to disparities in self-rated health status and preventive health behaviors in older adults. Ann Fam Med.

[37] Cho YI, Lee SYD, Arozullah AM, Crittenden KS (2008). Effects of health literacy on health status and health service utilization amongst the elderly. Soc Sci Med.

[38] Sudore RL, Mehta KM, Simonsick EM, Harris TB, Newman AB, Satterfield S, Rosano C, Rooks RN, Rubin SM, Ayonayon HN, Yaffe K (2006). Limited literacy in older people and disparities in health and healthcare access. J Am Geriatr Soc.

[39] Toci E, Burazeri G, Jerliu N, Sørensen K, Ramadani N, Hysa B, Brand H (2015). Health literacy, self-perceived health and self-reported chronic morbidity among older people in Kosovo. Health Promot Int.

[40] Brinda EM, Rajkumar AP, Attermann J, Gerdtham UG, Enemark U, Jacob KS (2016). Health, social, and economic variables associated with depression among older people in low and middle income countries: World Health Organization study on global AGEing and adult health. Am J Geriatr Psychiatry.

[41] Gama D, Colombo D (2010). Closing the gap in a generation: health equity through action on the social determinants of health. Final report of the commission on social determinants of health. Rev Direito Sanitário.

[42] Vaalavuo M (2016). Deterioration in health: what is the role of unemployment and poverty?. Scand J Public Health.

[43] Mahmood A, Chaudhury H, Michael YL, Campo M, Hay K, Sarte A (2012). A photovoice documentation of the role of neighborhood physical and social environments in older adults’ physical activity in two metropolitan areas in North America. Soc Sci Med.

[44] Zhang X, Dupre ME, Qiu L, Zhou W, Zhao Y, Gu D (2017). Urban-rural differences in the association between access to healthcare and health outcomes among older adults in China. BMC Geriatr.

[45] Albrecht GL, Devlieger PJ (1999). The disability paradox: high quality of life against all odds. Soc Sci Med.

[46] Kusumastuti S, Derks MGM, Tellier S, Di Nucci E, Lund R, Mortensen EL, Westendorp RGJ (2016). Successful ageing: a study of the literature using citation network analysis. Maturitas.

