# Supplementary material for: A multidimensional model of healthy ageing: proposal and evaluation of determinants based on a population survey in Ecuador
Source: BMC Geriatr. 2021 Nov 1;21:615. doi: 10.1186/s12877-021-02548-5 (PMC8559396; doi:10.1186/s12877-021-02548-5)
Supplement: Supplementary file 1 — Additional file 1 Theoretical model used for the classification into healthy and less healthy ageing groups. [file 12877_2021_2548_MOESM1_ESM.docx]

**Additional file**

**Additional file 1.** Theoretical model used for the classification into *healthy* and *less healthy ageing groups.*

| **Domain** | **Variable** | **Healthy ageing group** | **Less healthy ageing group** |
| --- | --- | --- | --- |
| Physical and metabolic health | Arterial hypertension | ● No hypertension  ● With a diagnosis of hypertension, but blood pressure within normal parameters; takes medication  ● With a diagnosis of hypertension, but blood pressure within normal parameters; does not take medication | ● With a diagnosis of hypertension and blood pressure is > 150/90 |
|  | Diabetes | ● Without diabetes  ● With an established diagnosis of diabetes, fasting glucose between 80-126 mg / dl, and takes medication | ● With an established diagnosis of diabetes |
|  | Cancer | - Without cancer | - With cancer |
|  | Cronic lung disease (COPD) | - Without COPD - With COPD, but does not limit his/her daily activities | - With COPD that limits his or her daily activities |
|  | Cardiovascular disease | - No heart problems - With heart problems, but does not limit his/her daily activities | - With heart problems that severely limit his or her daily activities |
|  | Cerebrovascular  disease | - No cerebrovascular problems - With cerebrovascular problems that limit his or her activities a little or not at all | - With cerebrovascular problems that severely limit his or her daily activities |
|  | Arthritis, Rheumatism,  Asteoarthritis | ● No arthritis  ● With arthritis that limit his or her activities a little or not at all | - Arthritis that severely limits his or her activities |
|  | Osteoporosis | - Without osteoporosis | - With osteoporosis |
| Geriatric syndroms | Urinary and fecal incontinence | - No urinary or fecal incontinence | - Urinary or fecal incontinence |
|  | Falls | - No falls syndrome (has not fallen) | - With falls syndrome (has fallen) |
|  | Polipharmacy | - No (use of 4 or fewer medications) | - Yes (consumption of 5 or more medications) |
| Risk factors | Arterial hypertension | - Blood pressure <150/90 | - Blood pressure >150/90 |
|  | Hyperglycemia | - Fasting glucose <126 | - Fasting glucose > 126 |
|  | Obesity | - Body mass index < 29 | - Body mass index > 30 |
|  | Hyperlipidemia | - Normal values of HDL, LDL and triglycerides | - Abormal values of HDL, LDL and triglycerides |
|  | Alcohol consumption | - No | - Yes |
|  | Tobbaco | - Never smoked | - Current consumption - Used to smoke |
|  | Physical activity | - Yes | - No |
| Cognitive ability | Cognitive impairment  (Mini-mental) | - Greater than or equal to 14 | - Less than or equal to 13 |
|  | Dementia | - Without dementia | - With a diagnosis of dementia |
| Physical capacity | Independence in activities of daily living (Katz Index) | - Absence of disability | ● With moderate or severe disability |
|  | Mobility | - Greater than or equal to 6 out of 7 to the following: stands with the feet together keeping eyes open, stands with the heel of one foot in front of the other foot, stands on one foot without leaning or holding onto anything, feels able to get up quickly from the chair five times, gets up quickly from the chair five times, feels able to get up from the chair with the arms on the chest five times, and gets out of the chair with the arms on the chest five times | - Less than or equal 5 to the following: stands with the feet together keeping eyes open, stands with the heel of one foot in front of the other foot, stands on one foot without leaning or holding onto anything, feels able to get up quickly from the chair five times, gets up quickly from the chair five times, feels able to get up from the chair with the arms on the chest five times, and gets out of the chair with the arms on the chest five times |
| Psychological well-being | Physical Abuse | - No | - Answers yes to any of the physical abuse questions |
|  | Sexual Abuse | - No | - Answers yes to any of the sexual abuse questions |
|  | Psychological Abuse | - No | - Answers yes to any of the psychological abuse questions |
|  | Depression (Yesavage Scale) | - Less than or equal to 4 (without depression) | - Greater than or equal to 4 (without depression) |
| Social welfare | Negligence | - No | - Answers yes to any of the negligence questions |
|  | Financial violence | - No | - Answers yes to any of the financial violence questions |
|  | Social risk (Gijón scale) | - Less than or equal to 9 | - Greater than or equal to 10 (risk) |
| Political environment | Support from the environment | - Receives support | - Does not receive support |
|  | Human development voucher (policies)^a^ | - Receives a “Human development voucher” | - Does not receive a “Human development voucher” |
|  | Access to Health services | - Yes | - No |
|  | Access to social security | - Yes | - No |
| Interactions with the environment | Community participation | ● Provides voluntary support to an institution or organization in the community at least once a day / week / month  ● Attends older adult groups, day centers, church, etc. | ● Does not provide support  ● Provides support 1 time a year, less than once a year, does not know, does not respond   - ● Does not attend older adult groups, day centers, church, etc. |
|  | Provides support | - Provides support to his or her children, siblings and other family or friends | - Does not provide support |
|  | Receive support | - Receives support from children, siblings and other family or friends | - Does not receive support |

^a^Human development voucher = Government financial aid given to people living in extreme poverty in Ecuador.
